# The impact of *ACE2* polymorphisms (rs1978124, rs2285666, and rs2074192) and *ACE1* rs1799752 in the mortality rate of COVID-19 in different SARS-CoV-2 variants

**DOI:** 10.1186/s40246-023-00501-8

**Published:** 2023-06-16

**Authors:** Farzaneh Sheikhian, Sahar Sadeghi Mofrad, Samira Tarashi, Morteza Ghazanfari Jajin, Fatemeh Sakhaee, Iraj Ahmadi, Enayat Anvari, Mojgan Sheikhpour, Abolfazl Fateh

**Affiliations:** 1 Department of Biology, Parand Branch, Islamic Azad University, Parand, Iran; 2 Department of Microbiology, Islamic Azad University of Central Tehran Branch, Tehran, Iran; 3 Department of Mycobacteriology and Pulmonary Research, Pasteur Institute of Iran, Tehran, Iran; 4 Microbiology Research Center (MRC), Pasteur Institute of Iran, Tehran, Iran; 5 Department of Physiology, School of Medicine, Ilam University of Medical Science, Ilam, Iran; 6 Clinical Research Development Unit, Shahid Mostafa Khomeini Hospital, Ilam University of Medical Science, Ilam, Iran

**Keywords:** SARS-CoV-2 variants, COVID-19, *ACE2* polymorphisms, *ACE1* rs1799752

## Abstract

**Background:**

Clinical severity of severe acute respiratory syndrome coronavirus 2 (SARS-CoV-2) outcomes could be influenced by genetic polymorphisms in angiotensin I-converting enzyme (*ACE1*) and *ACE2*. This study aims to examine three polymorphisms (rs1978124, rs2285666, and rs2074192) on the *ACE2* gene and *ACE1* rs1799752 (I/D) in patients who have coronavirus disease 2019 (COVID-19) with various SARS-CoV-2 variants.

**Methods:**

Based on polymerase chain reaction-based genotyping, four polymorphisms in the *ACE1* and *ACE2* genes have been identified in 2023 deceased patients and 2307 recovered patients.

**Results:**

The *ACE2* rs2074192 TT genotype was associated with the COVID-19 mortality in all three variants, whereas the CT genotype was associated with the Omicron BA.5 and Delta variants. *ACE2* rs1978124 TC genotypes were related to COVID-19 mortality in the Omicron BA.5 and Alpha variants, but TT genotypes were related to COVID-19 mortality in the Delta variant. It was found that *ACE2* rs2285666 CC genotypes were associated with COVID-19 mortality in Delta and Alpha variants, and CT genotypes in Delta variants. There was an association between *ACE1* rs1799752 DD and ID genotypes in the Delta variant and COVID-19 mortality, whereas there was no association in the Alpha or Omicron BA.5 variants. In all variants of SARS-CoV-2, CDCT and TDCT haplotypes were more common. In Omicron BA.5 and Delta, CDCC and TDCC haplotypes were linked with COVID-19 mortality. In addition to COVID-19 mortality, the CICT, TICT, and TICC were significantly correlated.

**Conclusion:**

The *ACE1*/*ACE2* polymorphisms had an impact on COVID-19 infection, and these polymorphisms had different effects in various SARS-CoV-2 variants. To confirm these results, however, more research needs to be conducted.

**Supplementary Information:**

The online version contains supplementary material available at 10.1186/s40246-023-00501-8.

## Introduction

Coronavirus disease 2019 (COVID-19), caused by the severe acute respiratory syndrome coronavirus 2 (SARS-CoV-2), has resulted in increased mortality and morbidity rates around the globe [1]. With millions of cases confirmed around the world, it will be helpful to know the risk factors and protective factors of COVID-19 infection for the prevention, progression, and unfavorable outcomes of COVID-19 infections. Researchers have identified several risk factors for COVID-19 among adults, including underlying illnesses such as cardiovascular disease, hypertension, and chronic obstructive pulmonary disease, in addition to demographics such as age, ethnicity, gender, and host genetics. Various studies in Iran have evaluated the association of gene polymorphisms involved in SARS-CoV-2 infection, such as interferon lambda 3 and 4 (rs12979860, rs12980275, rs8099917, and rs368234815), tripartite motif containing-22 (rs7113258, rs7935564, and rs1063303), vitamin D receptor gene (*ApaI*, *BsmI*, *BglI*, *TaqI*, *FokI*, and *Tru9I*), interferon-induced transmembrane-protein 3 (rs6598045 and rs12252), transmembrane serine protease 2 (rs12329760), interleukin-10 (rs1800871, rs1800872, and rs1800896), and *ABO* rs657152 [2–12]. Infection and development of COVID-19 can be prevented with healthy eating habits, adequate nutrition, and COVID-19 vaccination. It has been suggested that these factors can reduce inflammation through increased anti-inflammatory cytokines and decreased expression of angiotensin-converting enzyme-2 (*ACE2*) [1]. By reducing SARS-CoV-2 binding sites and limiting viral entrance into cells, decreasing *ACE2* expression would lead to people less susceptible to the virus. After binding the SARS-CoV-2 to ACE2, the expression of *ACE2* is downregulated. However, it should be noted that lack of the positive effects of *ACE2* may exacerbate lung injury through a number of different routes [13].

The SARS-CoV-2 enters the cells by the binding of the spike protein to the ACE2 receptor, a key actuator in the renin–angiotensin–aldosterone system (RAAS). Therefore, in COVID-19, RAAS has been directly linked to the development of acute respiratory distress syndrome (ARDS) as part of the host tissue response [14, 15]. However, these same drugs may also be helpful during the inflammation stage of the disease. *ACE*, *ACE2*, and *AT1R* expression has been shown to be altered by chronic treatment with ACE inhibitors [16, 17].

It has been hypothesized that single-nucleotide polymorphisms (SNPs) in the *ACE2* gene could affect both its expression and its binding affinity to SARS-CoV-2, affecting both COVID-19 severity and susceptibility to SARS-CoV-2 infection [18, 19]. One of the most extensively studied SNPs in *ACE2* is located in the splice region (rs2285666, G>A, Intron 3/4). It has been shown to be associated with coronary heart disease, hypertension, and diabetes associated with stroke [20, 21].

An *ACE* (17q23.3) is another commonly mentioned gene that produces angiotensin-converting enzyme. It converts angiotensin I (Ang I) to Ang II and activates the AT1R, which has vasoconstrictor, pro-inflammatory, and fibrotic properties [22]. It has also been suggested that COVID-19 may be affected by the deletion/insertion (D/I) of an Alu repeat at the *ACE* locus (rs1799752, Intron 16) [13]. The serum *ACE* levels of *ACE1* rs1799752 D-allele carriers are considerably higher than those of I-variant carriers. In addition to being associated with an elevated risk of myocardial infarction and left ventricular hypertrophy, the D-allele is a risk factor for onset and progression of ARDS and SARS [20, 23].

There is a possible role for *ACE2* rs1978124, located in the intron of chromosome Xp22, in affecting gene function. There is also a link between the *ACE2* rs1978124 and dyslipidemia, the risk of diabetes-related left ventricular remodeling, and the severity of COVID-19 [24, 25].

The *ACE2* rs2074192 is located in intron 16. Based on analysis of this SNP, rs2074192 is thought to alter donor location and protein features. It has been reported that it is associated with left ventricular hypertrophy [26].

As part of our previous study on the possibility of using a personalized approach in the selection of therapy for COVID-19 patients [3–11], we investigated four different SNPs that are relevant to the *ACE1* and *ACE2* genes and their haplotype combinations in predicting susceptibility to SARS-CoV-2 infection or COVID-19 severity. This prompted us to conduct a pilot study that examined the gene variants of *ACE2* rs1978124, rs2285666, and rs2074192 as well as *ACE1* rs1799752 (I/D) in individuals with different SARS-CoV-2 variants that were clinically divided into improved and deceased individuals.

## Materials and methods

### Sample collection

The study involved 4330 patients with COVID-19 recruited from a hospital in Ilam City, Kurdish region, between October 2020 and March 2022 during the three peaks of infection for COVID-19 (Omicron BA.5, Delta, and Alpha).

According to the inclusion criteria, only 4330 patients out of 13,450 cases were selected. There are several inclusion criteria, including being eager to participate in the study, Iranian nationality combined with a common ethnicity, no previous infection or vaccination against COVID-19, being selected from only one hospital, having a positive reverse transcription polymerase chain reaction test result, and no underlying comorbidities, including lung, heart, liver, kidney, cancer, pregnancy, immunocompromised diseases, and obesity, that COVID-19 infection severity could be affected by these factors.

By World Health Organization guidelines, 4330 participants were split into two groups: 2307 recovered COVID-19 patients (outpatients) as control group, and 2023 deceased patients of the same gender and age as case group. In the absence of healthy control subjects, we considered recovered patients with mild and moderate symptoms as controls while deceased patients (inpatients) with severe or critical symptoms were considered cases [27].

We obtained all clinical characteristics upon entering the hospital, including liver enzymes, lipid profiles, uric acid, creatinine, complete blood count (CBC), erythrocyte sedimentation rate (ESR), fasting blood glucose (FBS), C-reactive protein (CRP), 25-hydroxyvitamin D levels, and real-time PCR cycle threshold (Ct) values.

### *ACE2* SNPs and* ACE1* rs1799752 (I/D) genotyping

The total DNA of buffy coat samples was extracted according to manufacturer's instructions using a blood DNA extraction kit (Arman Gene Tajhiz Co.). Then, gel electrophoresis was performed and the DNAs were tested for quality and purity using NanoDrop spectrophotometers (Thermo Scientific, USA).

Genotyping of *ACE2* SNPs was performed by polymerase chain reaction–restriction fragment length polymorphism (PCR–RFLP). For *ACE2* rs1978124 polymorphism, PCR products of 553 bp were amplified using primers f-5′-CAACCACACATACCACAAT-3′ and r-5′-TTTCCTTTAGCCTACAATATCAAT-3′. An overnight treatment with one unit (U) of the *Echo471* (*Ava II*) (Thermo Fisher Scientific™, USA) restriction enzyme was then performed on the PCR product. After digestion, the sizes of the products were 464 and 89 bp for CC genotypes and 553 bp for TT genotypes [27].

To analyze the *ACE2* rs2285666 polymorphism, the f-5′-AAACCACTGAAATGACTTACTTACTG-3′ and r-5′-GCCTCACTGTCCTATGACTTTAT-3′ were used to produce 673 bp fragments. In a 16-h digest with 1 U of *AluI* (Fermentas, Vilnius, Lithuania), PCR products were digested at 37 °C [21]. The product size for TT genotypes was 177 and 496 bp, and for CC genotypes it was 673 bp.

To genotype *ACE2* rs2074192, in-house tetra-primer amplification refractory mutation system PCR method was used. With the help of the PRIMER1 website (http://primer1.soton.ac.uk/primer1.html), the outer forward (5′-AAGGGGACACTTAGACAAAATAAAA-3′), outer reverse (5′-GATCCAGAATGTTCTCCATATCAT-3′), inner forward (5′-GTGTGGAAATGTATAAATGGTTTGT-3′) and inner reverse (5′-ACAGCAGTCACAAATGAATAACTG-3′) primers were designed. In order to facilitate accurate differentiation between the two alleles, a mismatched nucleotide was added in the third position from the 3' end terminal of the primers. The result of rs2074192 genotypes was 777 bp + 379 bp for TT genotype and for CC genotype was 777 bp + 447 bp.

The *ACE1* rs1799752 intronic *Alu* insertion (I) or deletion (D) polymorphism was genotyped using PCR with specific primers (f-5′-CTGGAGACCACTCCCATCCTTTCT-3′ and r-5′-GATGTGGCCATCACATTCGTCAGAT-3′). In the DD genotype, the product was 191 bp, in the II genotype, 480 bp, and in the ID genotype, 480 bp + 191 bp [27].

For confirmation of PCR findings, at least 10% of the samples were randomly genotyped using the Sanger sequencing method on an ABI 3500 DX Genetic Analyzer (ABI, Thermo Fisher Scientific, MA, USA). Using Mega Version 11.0 (https://www.megasoftware.net/), the findings were assessed.

### Statistical analyses

Statistical analyses were directed using SPSS 22.0. (SPSS. Inc, Chicago, IL, USA). Continuous data were evaluated using the Mann–Whitney test, and numerical variables were reported as mean ± standard deviation (SD). SNP frequencies were expressed as numbers (%) in each group, and Chi-square test was used to analyze the results. The SNPStats software (http://bioinfo.iconcologia.net/SNPStats) was used to assess all genetic models, including dominant, recessive, co-dominant, and over-dominant models of inheritance for four SNPs. Using the Akaike Information Criterion (AIC) and the Bayesian Information Criterion (BIC), the best model was determined. Additionally, using SNPStats, haplotype frequencies were found for *ACE1* and *ACE2* based on the expectation maximization algorithm. The software SNPStats also determined the Hardy–Weinberg equilibrium (HWE) and the minor allele frequency (MAF). The odds ratios (ORs) and 95% confidence intervals (CIs) were calculated to estimate the strength of the association. The analysis was conducted using two-sided tests, and a *P* value ≤ 0.05 was considered statistically significant. Multiple comparisons were Bonferroni-corrected.

## Results

### General characteristics of patients

In this study, 4330 patients participated, including 1395 Alpha variant, 1425 Delta variant, and 1510 Omicron BA.5 variant. The mean age of patients with the Alpha, Delta, and Omicron BA.5 variants were 54.7 ± 12.5, 57.3 ± 12.1, and 52.4 ± 13.1, respectively, which was statistically significant. The frequency of deceased patients was 632 (45.3%), 913 (64.1%), and, 478 (31.7%) in the Alpha, Delta, and Omicron BA.5 variants, respectively.

The frequency of *ACE2* rs2074192 CC genotype in the Omicron BA.5 variant (54.9%) was higher than both variants and *ACE2* rs2074192 TT genotype in the Delta variant (35.1%) was higher than the Alpha (25.9%) and Omicron BA.5 variants (24.8%), which was statistically significant (*P* < 0.001) (Table 1).

**Table 1:** The frequencies of *ACE2* and *ACE1* polymorphisms between SARS-CoV-2 variants

Variables	SARS-CoV-2 variants			*P* value
	Alpha (n = 1395)	Delta (n = 1425)	Omicron BA.5 (n = 1510)	
Deceased/recovered patients	632/763 (45.3/54.7%)	913/512 (64.1/35.9%)	478/1032 (31.7/68.3%)	< 0.001*
Mean age ± SD	54.7 ± 12.5	57.3 ± 12.1	52.4 ± 13.1	< 0.001*
Gender (male/female)	735/660 (52.7/47.3%)	760/665 (53.3/46.7%)	765/745 (50.7/49.3%)	0.317
*ACE2* rs2074192				< 0.001*
CC	744 (53.3%)	637 (44.7%)	829 (54.9%)	
CT	290 (20.8%)	288 (20.2%)	307 (20.3%)	
TT	361 (25.9%)	500 (35.1%)	374 (24.8%)	
*ACE2* rs1978124				< 0.001*
TT	865 (62.0%)	918 (64.4%)	756 (50.1%)	
TC	196 (14.1%)	136 (9.6%)	446 (29.5%)	
CC	334 (23.9%)	371 (26.0%)	308 (20.4%)	
*ACE2* rs2285666				< 0.001*
CC	831 (59.6%)	850 (59.6%)	570 (37.7%)	
CT	323 (23.2%)	275 (19.6%)	747 (49.5%)	
TT	241 (17.2%)	300 (21.1%)	193 (12.8%)	
*ACE1* rs1799752 (I/D)				< 0.001*
DD	474 (34.0%)	539 (37.8%)	373 (24.7%)	
ID	627 (44.9%)	587 (41.2%)	778 (51.5%)	
II	294 (21.1%)	299 (21.0%)	359 (23.8%)	

SD, standard deviation; SARS-CoV-2, Severe Acute Respiratory Syndrome Coronavirus 2; *ACE2*, angiotensin-converting enzyme 2

*Statistically significant (< 0.05)

The frequency of *ACE2* rs1978124 TT, TC, and CC genotypes in different SARS-CoV-2 variants was tabulated in Table 1. It was statistically significant (*P* < 0.001) that the frequency of *ACE2* rs1978124 TT genotype in the Delta variant (64.4%) was higher than that in both variants, and the frequency of CC genotype in the Omicron BA.5 variant (20.4%) was higher than that in both variants of Alpha (23.9%) and Delta (26.0%).

According to Table 1, the frequency of *ACE2* rs2285666 CC genotype in Omicron BA.5 variants (37.7%) was lower than both variants, while the Delta variant's TT genotype (21.1%) was higher than that of Alpha (17.2%) and Omicron BA.5 (12.8%), which was statistically significant (*P* < 0.001).

### Relationship between *ACE2 *SNPs and *ACE1* rs1799752 (I/D) polymorphism and COVID-19 mortality adjusted by SARS-CoV-2 variants

**Fig. 1:**
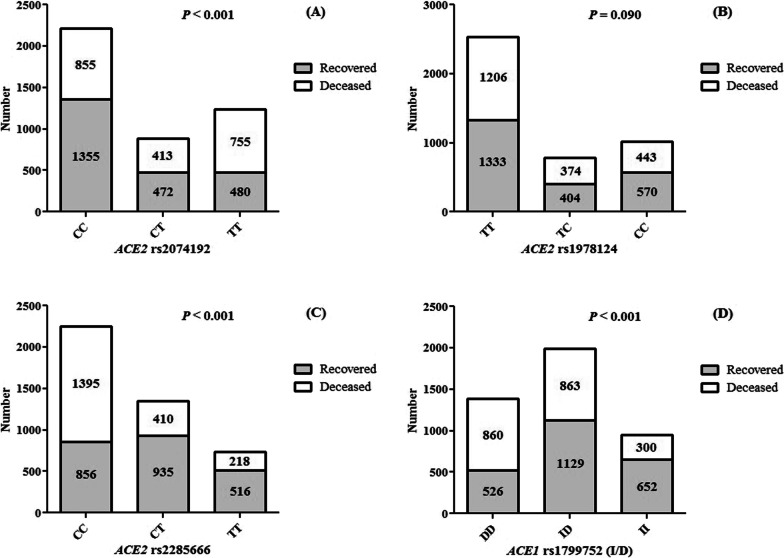
The frequencies of ACE2 rs2074192 (**A**), rs1978124 (**B**), rs2285666 (**C**) and ACE1 rs1799752 (I/D) (**D**) on recovered and deceased patients

As shown in Fig. 1, the mortality rate of COVID-19 was significantly higher in patients with *ACE2* rs2074192 TT (*P* < 0.001), *ACE2* rs2285666 CC (*P* < 0.001), and *ACE1* rs1799752 DD (*P* < 0.001) genotypes than in those with the other genotypes, while there was no relationship between *ACE2* rs1978124 polymorphism and rate of the COVID-19 mortality (*P* = 0.090).

Table 2 summarizes the results of the inheritance model analysis of *ACE2* SNPs and *ACE1* rs1799752 (I/D) polymorphisms in the patients. The best-fitting models for all *ACE2* SNPs and *ACE1* rs1799752 (I/D) were codominant heredity models with the lowest BIC and AIC values. It was shown that *ACE2* rs2074192 TT genotypes (*P* < 0.0001, OR 2.43, 95% CI 2.09–2.83) were associated with a higher risk of COVID-19 mortality. The *ACE2* rs1978124 TC genotype (*P* < 0.0001, OR 1.72, 95% CI 1.41–2.10), *ACE2* rs2285666 CC genotype, and *ACE1* rs1799752 D/D genotype were related to an increased risk of COVID-19 mortality.

**Table 2:** *ACE2* SNPs and *ACE1* rs1799752 (I/D) polymorphism association with COVID-19 mortality adjusted by SARS-CoV-2 variants

*ACE2* rs2074192		Groups		OR (95% CI)	*P* value	AIC	BIC
Model	Genotype	Recovered patients	Deceased patients				
Allele	C	3182 (69.0%)	2123 (52.0%)	–	–	–	–
	T	1432 (31.0%)	1923 (48.0%)	–	–	–	–
Codominant	C/C	1355 (58.7%)	855 (42.3%)	1.00	< 0.0001*	5542.5	5580.8
	C/T	472 (20.5%)	413 (20.4%)	1.60 (1.31–1.94)			
	T/T	480 (20.8%)	755 (37.3%)	2.43 (2.09–2.83)			
Dominant	C/C	1355 (58.7%)	855 (42.3%)	1.00	< 0.0001*	5558.0	5589.8
	C/T–T/T	952 (41.3%)	1168 (57.7%)	2.15 (1.87–2.47)			
Recessive	C/C–C/T	1827 (79.2%)	1268 (62.7%)	1.00	< 0.0001*	5562.3	5594.1
	T/T	480 (20.8%)	755 (37.3%)	2.14 (1.86–2.46)			
Overdominant	C/C–T/T	1835 (79.5%)	1610 (79.6%)	1.00	0.64	5677.1	5709.0
	C/T	472 (20.5%)	413 (20.4%)	1.04 (0.87–1.25)			
Minor allele frequency (T)	0.31	0.48	–	–	–	–	
*ACE2* rs1978124							
Allele	T	3070 (67.0%)	2786 (69.0%)	–	–	–	–
	C	1544 (33.0%)	1260 (31.0%)	–	–	–	–
Codominant	T/T	1333 (57.8%)	1206 (59.6%)	1.00	< 0.0001*	5639.2	5677.4
	T/C	404 (17.5%)	374 (18.5%)	1.72 (1.41–2.10)			
	C/C	570 (24.7%)	443 (21.9%)	0.85 (0.73–0.99)			
Dominant	T/T	1333 (57.8%)	1206 (59.6%)	1.00	0.26	5676.0	5707.9
	T/C–C/C	974 (42.2%)	817 (40.4%)	1.08 (0.95–1.23)			
Recessive	T/T–T/C	1737 (75.3%)	1580 (78.1%)	1.00	< 0.0001*	5666.1	5697.9
	C/C	570 (24.7%)	443 (21.9%)	0.78 (0.67–0.90)			
Overdominant	T/T–C/C	1903 (82.5%)	1649 (81.5%)	1.00	< 0.0001*	5641.3	5673.2
	T/C	404 (17.5%)	374 (18.5%)	1.80 (1.49–2.19)			
Minor allele frequency (C)	0.33	0.31	–	–	–	–	
*ACE2* rs2285666							
Allele	C	2647 (57.0%)	3200 (79.0%)	–	–	–	–
	T	1967 (43.0%)	846 (21.0%)	–	–	–	–
Codominant	C/C	856 (37.1%)	1395 (69.0%)	1.00	< 0.0001*	5306.8	5345.0
	C/T	935 (40.5%)	410 (20.3%)	0.33 (0.28–0.38)			
	T/T	516 (22.4%)	218 (10.8%)	0.23 (0.19–0.28)			
Dominant	C/C	856 (37.1%)	1395 (69.0%)	1.00	< 0.0001*	5315.5	5347.3
	C/T–T/T	1451 (62.9%)	628 (31.0%)	0.29 (0.25–0.33)			
Recessive	C/C–C/T	1791 (77.6%)	1805 (89.2%)	1.00	< 0.0001*	5666.1	5697.9
	T/T	516 (22.4%)	218 (10.8%)	0.33 (0.28–0.40)			
Overdominant	C/C–T/T	1372 (59.5%)	1613 (79.7%)	1.00	< 0.0001*	5662.7	5594.6
	C/T	935 (40.5%)	410 (20.3%)	0.46 (0.40–0.53)			
Minor allele frequency (T)	0.43	0.21	–	–	–	–	
*ACE1* rs1799752 (I/D)							
Allele	D	2181 (47.0%)	2583 (64.0%)	–	–	–	–
	I	2433 (53.0%)	1463 (36.0%)	–	–	–	–
Codominant	D/D	526 (22.8%)	860 (42.5%)	1.00	< 0.0001*	5481.3	5519.5
	I/D	1129 (48.9%)	863 (42.7%)	0.51 (0.44–0.59)			
	I/I	652 (28.3%)	300 (14.8%)	0.29 (0.24–0.35)			
Dominant	D/D	526 (22.8%)	860 (42.5%)	1.00	< 0.0001*	5523.8	5555.7
	I/D–I/I	1781 (77.2%)	1163 (57.5%)	0.43 (0.37–0.49)			
Recessive	D/D–I/D	1655 (71.7%)	1723 (85.5%)	1.00	< 0.0001*	5562.8	5594.6
	I/I	652 (28.3%)	300 (14.8%)	0.43 (0.37–0.50)			
Overdominant	D/D–I/I	1178 (51.1%)	1160 (57.3%)	1.00	0.0057*	5669.7	5701.5
	I/D	1129 (48.9%)	863 (42.7%)	0.84 (0.74–0.95)			
Minor allele frequency (I)	0.53	0.36	–	–	–	–	

COVID-19, coronavirus disease; *ACE2*, angiotensin-converting enzyme 2; OR, Odds ratios; CI, confidence intervals; AIC, Akaike information criterion; BIC, Bayesian information criterion; OR, odds ratios; CI, confidence intervals

*Statistically significant (< 0.05)

Based upon an adjustment for gender in *ACE2* rs2074192, the COVID-19 mortality rate was associated with TT (*P* < 0.0001, OR 2.28, 95% CI 1.79–2.09) and CT (*P* < 0.0001, OR 1.54, 95% CI 1.24–1.92) genotypes in female and TT genotype (*P* < 0.0001, OR 3.05, 95% CI 2.41–3.85) in male patients (Additional file 1: Table S1).

The results of gender adjustment for *ACE2* rs1978124 showed an association between the COVID-19 mortality rate and TC genotype (*P* < 0.0001, OR 1.70, 95% CI 1.38–2.09) in female and TT genotype (*P* < 0.0001, OR 1.28, 95% CI 1.08–1.52) in male patients (Additional file 1: Table 2).

As mentioned in supplementary Table 3, all genetic correlations were significant after Bonferroni correction.

*ACE2* SNPs and *ACE1* rs1799752 (I/D) polymorphisms were incompatible with HWE in both recovered and deceased individuals (*P* < 0.0001). It is important to note that these variants are not found in HWE, which may help to explain why they are associated with the COVID-19 infection.

### Distributions of *ACE2* SNPs and* ACE1* rs1799752 (I/D) polymorphism among SARS-CoV-2 variants

Different variants of the SARS-CoV-2 were closely linked to mortality rate. The Delta and Omicron BA.5 were associated with high and low mortality rates (*P* < 0.001), respectively.

All three COVID-19 variants were associated with the *ACE2* rs2074192 TT genotype after adjusting for SARS-CoV-2 variants with *ACE2* rs2074192 genotypes. As for patients infected with the Omicron BA.5 and Delta variants, *ACE2* rs2074192 CT genotype was associated with COVID-19 mortality, whereas the Alpha variant did not show the same association (Table 3).

**Table 3:** *ACE2* SNPs and *ACE1* rs1799752 (I/D) genotypes association with SARS-CoV-2 variants

Variants	rs2074192 Genotypes	Recovered patients	Deceased patients	OR (95% CI)
Alpha	C/C	437	307	1.00
	C/T	162	128	–
	T/T	164	197	1.79 (1.39–2.31)
Delta	C/C	314	323	1.00
	C/T	98	190	2.16 (1.59–2.94)
	T/T	100	400	3.94 (3.01–5.16)
Omicron BA.5	C/C	604	225	1.00
	C/T	212	95	1.39 (1.02–1.89)
	T/T	216	158	2.06 (1.59–2.67)

Variants	rs1978124 Genotypes	Recovered patients	Deceased patients	OR (95% CI)
Alpha	T/T	484	381	1.00
	T/C	83	113	2.08 (1.50–2.88)
	C/C	196	138	–
Delta	T/T	302	616	1.00
	T/C	74	62	–
	C/C	136	235	–
Omicron BA.5	T/T	547	209	1.00
	T/C	247	199	2.62 (2.00–3.43)
	C/C	238	70	–

Variants	rs2285666 Genotypes	Recovered patients	Deceased patients	OR (95% CI)
Alpha	C/C	299	532	1.00
	C/T	295	28	–
	T/T	169	72	–
Delta	C/C	265	585	1.00
	C/T	20	255	5.77 (3.58–9.30)
	T/T	227	73	–
Omicron BA.5	C/C	292	278	1.00
	C/T	620	127	–
	T/T	120	73	–

Variants	rs1799752 (I/D) Genotypes	Recovered patients	Deceased patients	OR (95% CI)
Alpha	D/D	216	258	1.00
	I/D	352	275	–
	I/I	195	99	–
Delta	D/D	152	387	1.00
	I/D	178	409	0.25 (0.19–0.34)
	I/I	182	117	–
Omicron BA.5	D/D	158	215	1.00
	I/D	599	179	–
	I/I	275	84	–

SARS-CoV-2, Severe Acute Respiratory Syndrome Coronavirus 2; *ACE2*, angiotensin-converting enzyme 2; OR, odds ratios; CI, confidence intervals

As a result of adjusting SARS-CoV-2 variants with *ACE2* rs1978124 genotypes, only the *ACE2* rs1978124 TC genotype in the Omicron BA.5 and Alpha variants was related to the mortality of COVID-19, whereas the TT genotype in the Delta variant was related to COVID-19 mortality (Table 3).

When SARS-CoV-2 variants were adjusted for *ACE2* rs228566 genotypes, there was an association between the *ACE2* rs228566 CC genotype in Delta and Alpha variants and the COVID-19 mortality. Additionally, *ACE2* rs2285666 CT genotypes are associated with COVID-19 mortality in patients with Delta variants, but not with Omicron BA.5 variants (Table 3).

As shown in Table 1, the frequency of *ACE1* rs1799752 DD genotype in the Delta variant (37.8%) was higher than both variants and II genotype in the Omicron BA.5 variant (23.8%) was higher than the Alpha (21.1%) and Delta variants (21.0%), which was statistically significant (*P* < 0.001).

A relationship between COVID-19 mortality and the *ACE1* rs1799752 DD and ID genotype was found in the Delta variant after adjusting SARS-CoV-2 variants for *ACE1* rs1799752 (I/D) genotypes, whereas this relationship did not exist in Alpha and Omicron BA.5 variants (Table 3).

### Haplotype analysis

Haplotype analysis was conducted using *ACE2* rs2074192, rs1978124, rs2285666 and *ACE1* rs1799752 (I/D), and ten significant haplotypes were identified. In all SARS-CoV-2 variants, CDCT and TDCT haplotypes were more common. In Delta and Omicron BA.5 variants, CDCC and TDCC haplotypes were associated with COVID-19 mortality. As reported in Table 4, CICT, TICT, and TICC all showed significant associations with COVID-19 mortality.

**Table 4:** SARS-CoV-2 variants and *ACE2* SNPs and *ACE1* rs1799752 (I/D) haplotypes

Haplotypes	Frequency	Alpha	Delta	Omicron
		OR (95% CI)	OR (95% CI)	OR (95% CI)
CDCT	0.2205	1.00	2.62 (1.74–3.96)	1.16 (0.77–1.75)
TDCT	0.15	1.41 (1.11–1.79)	5.36 (3.67–7.83)	1.34 (0.92–1.96)
CDCC	0.1042	–	2.63 (1.80–3.85)	1.18 (0.80–1.73)
TITT	0.074	–	2.34 (1.56–3.51)	–
TDCC	0.0697	–	4.02 (2.55–6.33)	1.73 (1.11–2.71)
CICT	0.0552	3.83 (2.45–6.00)	1.80 (1.12–2.92)	–
TITC	0.0419	–	2.36 (1.46–3.81)	–
TICT	0.0346	3.54 (2.07–6.06)	5.69 (2.32–13.94)	–
CICC	0.0247	3.08 (1.78–5.35)	–	–
TICC	0.0163	8.85 (3.55–22.07)	3.70 (1.28–10.66)	–

SARS-CoV-2, Severe Acute Respiratory Syndrome Coronavirus 2; *ACE2*, angiotensin-converting enzyme 2; SNPs, single nucleotide polymorphisms; OR, odds ratios; CI, confidence intervals

## Discussion

This comprehensive study evaluated the several SNPs located on *ACE1* rs1799752 (I/D) and *ACE2* (rs1978124, rs2285666, and rs2074192) genes in a cohort of 4330 Iranian patients positive for different SARS-COV-2 variants, divided into 2307 recovered and 2023 deceased COVID-19 patients.

There was a significant increase in COVID-19 mortality in patients with the *ACE2* rs2074192 T allele in this study. The T allele as MAF in the studied patients was 0.39, and it was lower in the recovered patients (0.31) than in the deceased patients (0.48). Other regions in which MAF is found in the NCBI dbSNP database include Asians (0.421), South Asians (0.239), East Asians (0.425), other Asians (0.400), Africans (0.316), African Americans (0.315), Europeans (0.446), and Latin Americans (0.419) (https://www.ncbi.nlm.nih.gov/snp/rs2074192).

In the current study, the *ACE2* rs2074192 TT genotype was related to COVID-19 mortality in all three variants. Additionally, the *ACE2* rs2074192 CT genotype was associated with COVID-19 mortality in Omicron BA.5 and Delta variant-infected patients, while it was not observed in Alpha variant-infected patients. It is suggested that the T allele is significantly more common in the symptomatic group of the rs2074192 SNP in both females and males as compared to the asymptomatic ones [28]. The findings of this study are also consistent with the findings of a recent large analysis of 1644 COVID-19 patients from the UK Biobank, which showed that the T allele is associated with worse outcomes from SARS-COV2 infection [29].

COVID-19 severity is associated with comorbidities such as cardiovascular risk, retinopathy in people with hypertension, type-2 diabetes, and hypertensive left ventricular hypertrophy in people with the T variant of the rs2074192 polymorphism [26, 30].

It is known that circulating Ang (1–7) levels in hypertensive females can be influenced by the rs2074192 polymorphism [31]. Since this SNP is intronic, it is unlikely to have an impact on the ACE2 protein sequence and, consequently, the effectiveness of the binding between the SARS-CoV-2 Spike protein and the ACE2 receptor. It is necessary to conduct functional studies to validate the effect of this variant on the expression of the ACE2 receptor and the accessibility of the receptor for SARS-COV-2 infection [32].

In this study, mortality rate of COVID-19 was significantly higher among patients with the T allele of *ACE2* rs1978124. According to the data, the T allele frequency was 0.68. This amount in the other regions was identified in Asian (0.20), South Asian (0.78), East Asian (0.14), other Asian (0.30), African (0.261), African American (0.261), European (0.625), and Latin American (0.744) (https://www.ncbi.nlm.nih.gov/snp/rs1978124).

This study showed that the TC genotype of *ACE2* rs1978124 in the Omicron BA.5 and Alpha variants was in association with COVID-19 mortality, whereas the TT genotype in the Delta variant was in association with COVID-19 mortality. As well, TC genotype was associated with a lower COVID-19 mortality rate in females and TT genotype with a higher COVID-19 mortality rate in males after adjusting for gender. SNPs rs2074192 and rs1978124 have protective effects on females based on genotype frequencies. Notably, the *ACE2* gene is on the X chromosome, making heterozygosity in males impossible.

Due to this, SNPs with their single copy may cause the worst outcomes in males [33]. In a study involving females, heterozygosity for the *ACE2* rs1978124 was associated with disease severity and it counts as a protective factor in females [25]. It also found that the *ACE2* rs1978124 T allele was independently correlated with higher mortality among people with acute coronary syndrome [34].

In this study, COVID-19 mortality was significantly higher in patients with the *ACE2* rs2285666 C allele, which is consistent with previous studies [20, 21, 35]. The frequency of the C allele was 0.52. This amount in the other regions was identified in Asian (0.446), South Asian (0.494), East Asian (0.438), other Asian (0.480), African (0.768), African American (0.765), European (0.796), and Latin American (0.624) (https://www.ncbi.nlm.nih.gov/snp/rs2285666).

COVID-19 mortality was associated with the *ACE2* rs2285666 CC genotype in the Alpha and Delta variants of this study. Additionally, the *ACE2* rs2285666 CT genotype was associated with COVID-19 mortality in Delta variant patients, but not with Omicron BA.5. Several reports indicate that the CC genotype at the *ACE2* rs2285666 increases the risk of contracting SARS-CoV-2. It was not possible to determine how this SNP affected COVID-19 severity or the COVID-19 mortality rates [36, 37]. In contrast to similar studies, this study examined a larger number of samples and included a different variant of SARS-CoV-2 than comparable studies. It should be noted that the racial factor should not be ignored because the results of the studies conducted in Iran were similar to our study [21, 35]. Also, in an Italian cohort indicated that there is no consistent correlation of *ACE2* variants with COVID-19 severity. It has speculated that rare susceptibility or resistant genotypes could be located in the non-coding regions of *ACE2* gene, known to play an important role in regulation of the gene activity [38].

The results of this study revealed that the *ACE1* rs1799752 DD and ID genotypes in the Delta variant were associated with COVID-19 mortality; however, the relationship was not found in the Alpha or Omicron BA.5 variants. The *ACE1* rs1799752 D-alleles are thought to affect how the SARS-CoV-2 entry receptor is expressed in the alveoli, and consequently how well it can infiltrate and cause disease [39].

Evidence suggests a negative correlation between the prevalence of the *ACE1* rs1799752 D allele and COVID-19 incidence. Furthermore, the frequency of the D allele was strongly associated with COVID-19-related mortality [40]. A review of *ACE1* rs1799752 (I/D) polymorphisms indicates that individuals with the DD genotype of COVID-19 may suffer severe lung damage [41].

Because *ACE1* rs1799752 (I/D) and *ACE2* rs1978124 are located in non-coding regions, they are unlikely to be functional polymorphisms. According to earlier research, the *ACE1* rs1799752 (I/D) polymorphism alters the balance of ACE1/ACE2, affecting serum and tissue levels of ACE1 protein. Approximately 50% of ACE function is attributed to the *ACE1* rs1799752 (I/D) polymorphism. Inflammation can be exacerbated by increased ACE activity, which increases angiotensin II levels [42].

This study offers new insight into the effect of polymorphisms in the *ACE1*/*ACE2* genes on COVID-19 mortality rates caused by different SARS-CoV-2 variants. There is still much to learn about the different behavior of different genotypes of these genes towards different SARS-CoV-2 variants, which deserves greater research and attention.

Several limitations accompanied this study. The frequency of these polymorphisms among healthy people without a previous history of COVID-19 infection could not be compared to that among patients due to the lack of healthy individuals with no history of COVID-19 infection. The absence of measuring serum *ACE* levels was another limitation of this study. Moreover, since this study was conducted among an ethnic group in Iran, more studies should be conducted with different ethnic groups to confirm these findings.

In conclusion, the finding of this study showed that the *ACE2* rs2074192 TT genotype in all three variants, the *ACE2* rs2074192 TC genotype in the Alpha and Omicron BA.5 variants and TT genotype in the Delta variant, the *ACE2* rs2285666 CC genotype in the Alpha and Delta variants and *ACE2* rs2285666 CT genotype in patients infected with the Delta variant was associated with the COVID-19 mortality. Also, the *ACE1* rs1799752 DD and ID genotypes in the Delta variant were associated with the COVID-19 mortality, while this relationship was not found in the Alpha and Omicron BA.5 variants. In order to confirm the results of this study, more research should be conducted in other places.

## Supplementary Information

**Additional file 1. Table S1:** ACE2 rs2074192 and sex cross-classification interaction. **Table 2:** ACE2 rs1978124 and sex cross-classification interaction. Table S3: Bonferroni-corrected test for all SNPs.

## Data Availability

All data generated or analyzed during this study are included in this published article.
