# Supplementary material for: The impact of ACE2 polymorphisms (rs1978124, rs2285666, and rs2074192) and ACE1 rs1799752 in the mortality rate of COVID-19 in different SARS-CoV-2 variants
Source: Hum Genomics. 2023 Jun 16;17:54. doi: 10.1186/s40246-023-00501-8 (PMC10273585; doi:10.1186/s40246-023-00501-8)
Supplement: Supplementary file 1 — Additional file 1. Table S1: ACE2 rs2074192 and sex cross-classification interaction. Table 2: ACE2 rs1978124 and sex cross-classification interaction. Table S3: Bonferroni-corrected test for all SNPs. [file 40246_2023_501_MOESM1_ESM.docx]

**Supplementary Table 1:** *ACE2* rs2074192 and sex cross-classification interaction

|  | **Female** | | | **Male** | | |
| --- | --- | --- | --- | --- | --- | --- |
| **Genotype** | **Recovered patients** | **Deceased patients** | **OR (95% CI)** | **Recovered patients** | **Deceased patients** | **OR (95% CI)** |
| CC | 403 | 228 | 1.00 | 952 | 627 | 1.20 (0.98-1.46) |
| CT | 472 | 413 | **1.54 (1.24-1.92)** | 0 | 0 | **-** |
| TT | 246 | 308 | **2.28 (1.79-2.09)** | 234 | 447 | **3.05 (2.41-3.85**) |

*ACE2*: angiotensin-converting enzyme 2; OR: Odds ratios; CI: confidence intervals.

**Supplementary Table 2:** *ACE2* rs1978124 and sex cross-classification interaction

|  | **Female** | | | **Male** | | |
| --- | --- | --- | --- | --- | --- | --- |
| **Genotype** | **Recovered patients** | **Deceased patients** | **OR (95% CI)** | **Recovered patients** | **Deceased patients** | **OR (95% CI)** |
| TT | 491 | 416 | 1.00 | 842 | 790 | **1.28 (1.08-1.52)** |
| TC | 404 | 374 | **1.70 (1.38-2.09)** | 0 | 0 | **-** |
| CC | 226 | 159 | **-** | 344 | 284 | 1.12 (0.90-1.38) |

*ACE2*: angiotensin-converting enzyme 2; OR: Odds ratios; CI: confidence intervals.

**Supplementary Table 3:** Bonferroni-corrected test for all SNPs

| **Group** | ***P*-value** | **Adjusted FDR method** | **Adjusted Bonferroni Method** |
| --- | --- | --- | --- |
| *ACE2* rs2074192 CT | 0.0023 | 0.0056 | 0.0276 |
| *ACE2* rs2074192 TT | 0.0051 | 0.0061 | 0.0612 |
| *ACE2* rs2074192 CC | 0.0051 | 0.0061 | 0.0612 |
| *ACE2* rs1978124 TC | 0.09 | 0.09 | 0.8900 |
| *ACE2* rs1978124 TT | 0.0024 | 0.0056 | 0.0288 |
| *ACE2* rs1978124 CC | 0.0012 | 0.0056 | 0.0144 |
| *ACE2* rs2285666 CC | 0.0049 | 0.0061 | 0.0588 |
| *ACE2* rs2285666 CT | 0.0022 | 0.0056 | 0.0264 |
| *ACE2* rs2285666 TT | 0.0033 | 0.0057 | 0.0035 |
| *ACE1* rs1799752 (I/I) | 0.0032 | 0.0056 | 0.0384 |
| *ACE1* rs1799752 (I/D) | 0.0033 | 0.0056 | 0.0396 |
| *ACE1* rs1799752 (D/D) | 0.0023 | 0.0056 | 0.0276 |
